# American Gynæcological Society

**Published:** 1888-10

**Authors:** 


### AMERICAN GYNÆCOLOGICAL SOCIETY.

September 18.

The President, Dr. Robert Battey, of Rome, called the Society to order.

Professor Fordyce Barker moved to reconsider the action which had been taken at the last meeting, and to accept the report of the committee which recommended joining the Congress of American Physicians and Surgeons.

This was seconded and carried.

Professor Barker was appointed a committee to inform the executive committee of the Congress of this action of the Society.

Dr. Howard A. Kelly, of Philadelphia, read a paper on "Ureteritis, its Diagnosis and Symptomatology."

Catheterization of the ureters is a proceeding which is very useful in the diagnosis of disease of the ureters and the pelvis of the kidneys. Ureteritis is probably more common than has usually been supposed. While this method of procedure is of comparatively recent date the disease—ureteritis—was recognized years ago by Rayer and by Cruveilhier. It may be either descending or ascending; perhaps it is more commonly secondary and descending. It may also be ascending, following gonorrhœa and other diseases which affect the bladder. The ureter may also be diseased from the passage of renal calculi, and as a complication of many forms of disease of the uterus and its surroundings. Hence it is important that the functions and the condition of the ureters should be carefully ascertained in any suspicious case. These and other facts suggest that more careful study of renal and bladder troubles is now in order for scientific gynæcologists.

Examination of the ureters may be made by inspection, by palpation, and by catheterization. The first may be practiced by splitting the vesico-vaginal septum at the proper level, then turning the opening into the vagina, as suggested by Dr. Thomas Addis Emmet. This may be done by pressure from without, or by pressure through the rectum, as suggested by Dr. Polk. By palpation the course of the ureter is to be followed by delicate touch, compression being exercised both through the abdominal wall and the vagina. If the ureter is dilated, its course may not infrequently be traced by this means. The method of catheterization, is however, the most practical, and the method of Pawlik, that of freehand catheterization, is believed to be the most applicable.

In three cases of supposed bladder trouble he found that it was the diseased ends of the ureters which were causing trouble, the trouble disappearing when these were cured. Other cases were also narrated to prove the value of this method of procedure. The literature of this subject is not extensive and includes, in addition to the papers of Pawlik and Simon, the theses of Bonnet and Chaumont, and the recent paper of Silbermann.

Dr. POLK found the subject one of great interest, and had given much time to its consideration, both upon the living and the dead subject. The method of examining the ureters which was most commonly referred to was Pawlik's. He had made many trials of it, but usually without much success. He had found more satisfaction in making a button-hole fistula at the base of the bladder as recommended by Dr. T. A. Emmet, and then by suitable pressure the ends of the ureters could be made accessible. In palpating for the ureter it is to be remembered that it is between the line of the uterus and the brim of the pelvis. For a catheter he preferred one of broad curve like a prostatic catheter, and after this had been entered another instrument should be passed into the rectum, with which the onward course of this instrument in the ureter could be traced.

Dr. H. T. BYFORD, of Chicago, had practiced palpation of the ureters in one hundred cases, desiring to find a simple method of determining its course. If the uterus is taken as a centre and the finger in the vagina be made to radiate from it along the pelvic roof, a line would at length be reached where a gentle resistance of a cord-like body would indicate the presence of the ureter. This line having been determined, it would not be difficult after a little practice to find the mouth of the ureter and introduce a catheter, the catheter being guided by the finger in the vagina.

Dr. W. H. BAKER, of Boston, thought that very few obtained sufficient skill to catheterize the ureters. He believed the method was of value, however, in enabling one to give a diagnosis as to the extent of diseases of the bladder, etc. It must not be forgotten, in all these manipulations, that there might be displacement of the ureter as had occurred in his experience.

Dr. H. C. COE, of New York, found in three autopsies that pressure was exercised upon the ureters two inches from their endings by cicatricial nodules which were associated with uterine disease. This condition might have caused dilatation of the ureters which would have admitted of their determination by palpation.

Dr. BACHE EMMET had found that as good results as by any method could be obtained as to determining the position of the ends of the ureters, by making a fistula in the base of the bladder, then pressing in the median line of the abdomen, then varying the pressure to one and then the other side. He believed that ureteral trouble complicated various uterine troubles, and that a diagnosis of pelvic disease could hardly be considered complete until the condition of the ureters was known. He did not believe that disease of the ureters was a very common complication of bladder disease.

Dr. KELLY, in closing, detailed the experiments of Tuchmann, Silbermann, and others in catheterization, and showed the value of the operation. He thought disease of the ureters could not be called uncommon, that it might be due to inflammatory deposits, displacements, etc., and that examination of these organs was eminently simple and practical.

Professor THOMAS ADDIS EMMET, of New York, presented a paper entitled "Cause and Treatment of Urethrocele."

This condition is generally considered as due to a loss of support in consequence of laceration of the perineum, but it is never found after first labors unless the rupture has extended through the sphincter ani. It always exists after serious double laceration of the cervix in consequence of severe labor, which may have been either rapid or tedious. The former is the more common cause, the latter sometimes acts as a cause when ergot has been given or when the labor has been instrumental. The trouble is usually in the lower or vaginal part of the urethra. In some cases the accident is doubtless prevented when a few ounces of urine are in the bladder during the expulsion of the head, the urine acting as a cushion and only being discharged as the space is required. This is contrary to the long accepted dictum that the bladder must be empty when the head is delivered. The laceration may be transverse or longitudinal, and a few fibres or many may be ruptured. There is seldom a rupture through the vaginal mucous membrane. Urethrocele must be differentiated from the thickened condition of the urethra in which there is no rupture. In urethrocele the length of the urethra is diminished, a pouch being formed in which the urine collects. Serious reflex symptoms may be excited by such a condition as is shown in a very aggravated case narrated in the paper.

Dr. PRIESTLY, of London, referred to a paper written by him in 1869, in which he referred to sebaceous cysts of the urethra which he thought formed a condition somewhat analogous to that referred to by Dr. Emmet in his paper. He thought such conditions as Dr. Emmet treated of might be due to other causes than labor; for example, a sebaceous cyst of the urethra which he had seen had arisen entirely independently of labor.

Professor SKENE, of Brooklyn, thought that cystocele and urethrocele were very apt to be confounded. In his experience it was not the lower portion of the urethra which was apt to be involved as the result of severe labor. Complete dislocation of the urethra, the latter being torn from its attachments at the pubic arch, was a more common accident of labor than the sacculated condition to which Dr. Emmet referred, and he believed the longitudinal or transverse ruptures of the fibres of the urethra to be relatively rare. The buttonhole operation would be of no use in cases of dislocated urethra, but for the sacculated condition it would apply. Bozeman had described the dilated condition of the urethra following labor ten or fifteen years ago, and had suggested the opening of the urethra as a means of relief.

Dr. LUSK, of New York, had found Emmet's operation very efficient in a very troublesome case which had long been under his observation, and had, finally, been cured by Dr. Emmet. Since then he had performed the operation in several cases with great satisfaction.

Dr. KELLY referred to a case of urethrocele which had been reported by Dr. Skene Keith, and upon which he had performed Emmet's operation with marked success. Urethrocele should be differentiated from false urethrocele or pouting of the urethra, which was often caused by straining in urination.

---

